# The international mobility of UK students; a government funded initiative

**DOI:** 10.1186/1757-1146-3-S1-O16

**Published:** 2010-12-20

**Authors:** Keith McCormick, Cathy Bowen, Jasper Tong, Mike Potter

**Affiliations:** 1 University of Southampton, Hampshire, UK,; 2 Singapore General Hospital, Sing Health Group, Singapore

## Introduction

The British Council Connect initiative was set up to support strategic alliances and partnerships, promoting student learning overseas. With suitable training, risk analysis and agreements in place, this gave us the potential to explore off shore practice based learning, clinical attachments.

## Method

A successful grant application was made to the British Council as part of the Prime Minister's Initiative for International Education (PMI2). A remote clinical educators network was identified, and training delivered. Five, third year students spent a month ‘hands on’ placement in five different hospitals in Singapore. Qualitative and quantitative data was collected from educators and students.

## Results

16 applicants competed for five places. All students passed their clinical attachment without incident, Overall mean rating for the placement; 5 out of 5 (1= poor and 5 = excellent). Mean student score for the value of ‘clinical activities’; 4.8. Overall mean student score for all 14 criteria; 4.51. Accommodation, induction and preparation for the placement; although satisfactory, were identified as areas for improvement.

## Discussion

This is the first UK podiatry initiative providing undergraduate students with hands on credit bearing clinical attachments overseas. Repeat funding is being sought. Students benefited greatly from high quality clinical education and the cultural experience.

